# Congenital Constriction Band With Lymphedema in a Neonate: A 17-Year Follow-Up After Single Stage Excision and Z-Plasty

**DOI:** 10.7759/cureus.15026

**Published:** 2021-05-14

**Authors:** Levin Kesu Belani, Abdul Halim Abd Rashid, Sharaf Ibrahim, Ahmad Fazly Abd Rasid, Muhd Kamal Muhd Abdul Jamil

**Affiliations:** 1 Orthopaedic & Traumatology, Fakulti Perubatan, Universiti Kebangsaan Malaysia, Kuala Lumpur, MYS; 2 Faculty Perubatan, Universiti Kebangsaan Malaysia Medical Centre, Kuala Lumpur, MYS; 3 Paediatric Orthopedics, Hospital Pakar Kanak-Kanak Universiti Kebangsaan Malaysia, Kuala Lumpur, MYS; 4 Paediatric Orthopaedics, Hospital Pakar Kanak-Kanak Universiti Kebangsaan Malaysia, Kuala Lumpur, MYS; 5 Paediatric Orthopaedics, Universiti Kebangsaan Malaysia Medical Centre, Kuala Lumpur, MYS

**Keywords:** z-plasty, single stage reconstruction, congenital anomalies of the lower extremity, congenital constriction band syndrome, long-term outcome

## Abstract

Congenital constriction band syndrome is a rare condition that presents with constriction bands affecting different types of extremities. Timely surgical intervention for moderate and severe stages of this condition can be performed either in a single stage or multiple stages. We report the case of a neonate who presented with a congenital constriction band and had excision of the constriction band and z-plasty reconstruction. The surgery was done in a single stage. This case highlights the outcome and safety of this single-stage surgery with Z-plasty reconstruction. At 17 years of age, he has a functional lower limb and excels in archery.

## Introduction

Congenital constriction band is a condition that can potentially be limb-threatening. There are to date 34 other terminologies describing this condition which can be confusing [1]. This condition is rare with a prevalence of 0.9 per 10000 births, consisting of 12% of all congenital upper limb deformities and 14% of all congenital lower limb deformities [2].

A differential diagnosis of this condition would be hair thread tourniquet syndrome which is another surgical emergency [3]. The surgical approach for this condition could be single-stage excision or multiple-stage excision [4]. We report the case of a neonate who was treated with a single-stage excision and followed up until skeletal maturity.

## Case presentation

A neonate was referred to our tertiary hospital at 14 days after birth with congenital constriction bands of the lower limb. The antenatal and birth history were uneventful.

**Figure 1:**
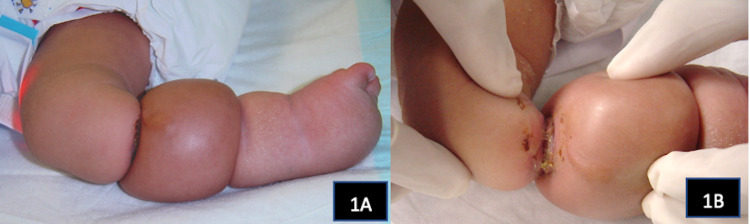
Double constriction bands causing lymphoedema of the right lower limb (1A). A close-up view showing full thickness skin loss at the site of the proximal constriction ring (1B).

Physical examination revealed an oedematous right lower limb and 2 constriction bands over the leg (Figure 1A,1B). The vascularity of the foot was normal with a capillary refill time of less than 2 seconds. He also had acrosyndactyly of the right hand and an in-utero amputation of the left index finger.

**Figure 2:**
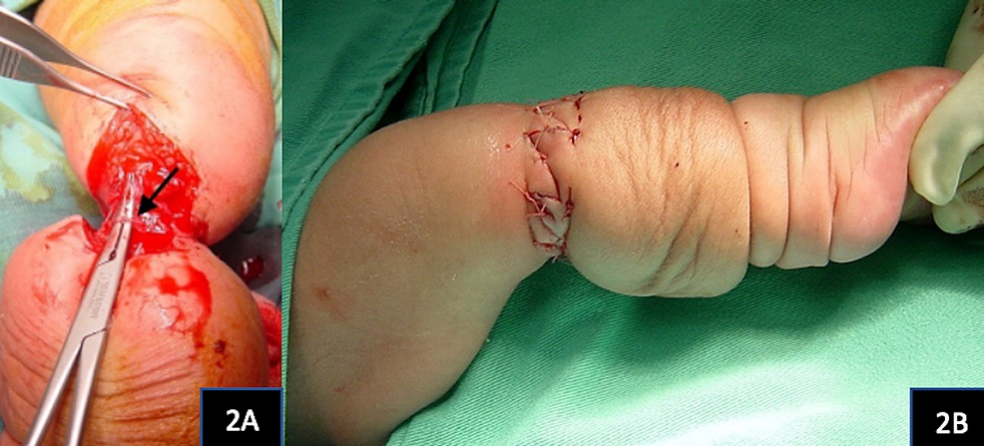
A deep constricting fibrous band (arrow) of the proximal ring was excised (2A). The skin was closed with z-plasties. There was immediate reduction of the oedema in the leg (2B).

An emergency single-stage excision of the proximal constriction band including the underlying deep fascia was done(Figure 2A). The skin was approximated via Z-plasty technique (Figure 2B). 

**Figure 3:**
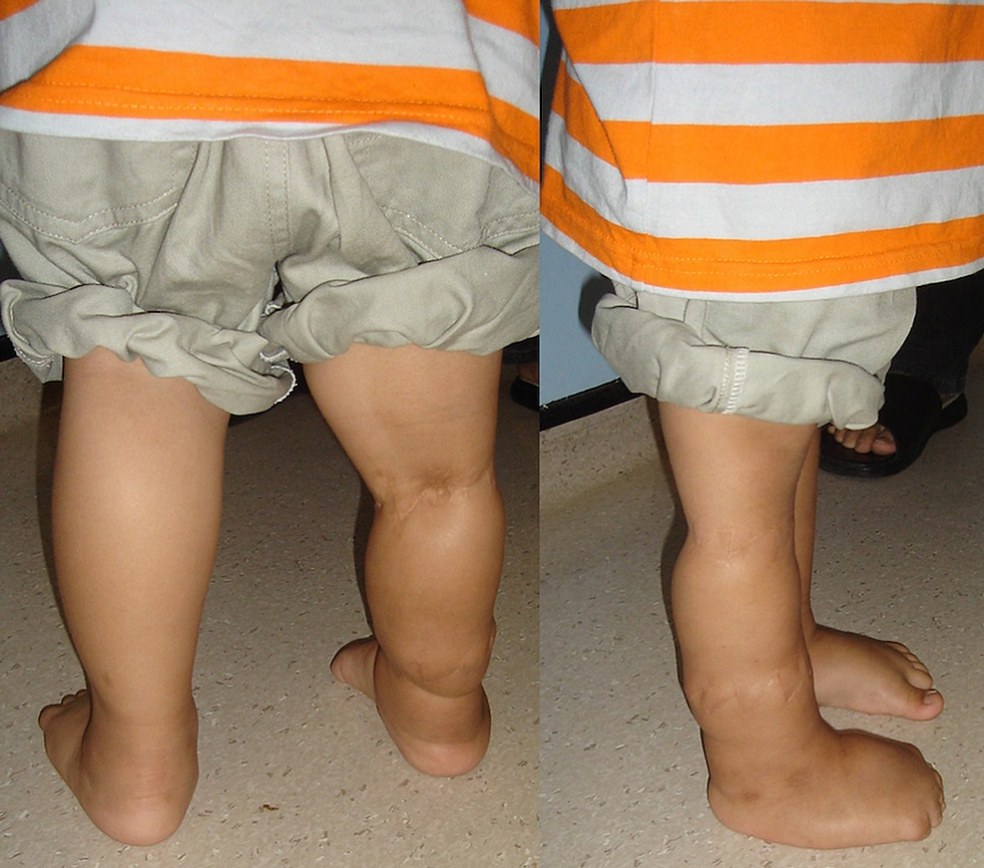
The photograph of both legs at the age of 2 years showing resolution of the leg oedema

Follow-up after two months showed the wound had healed and there was further reduction of the leg oedema. At one year of age, the distal constriction band was excised and the skin reconstructed via z-plasty technique . Follow-up at two years of age revealed well-healed scars and resolution of the leg oedema (Figure 3).

**Figure 4:**
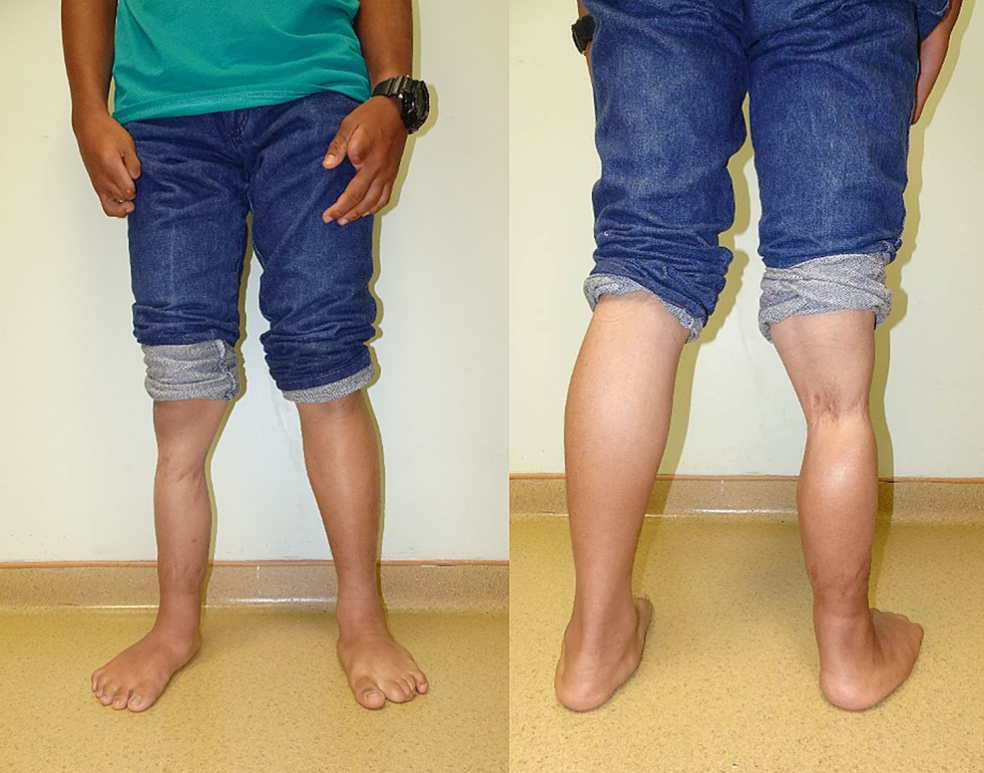
The photograph of both legs at the age of 17 years. He has no restriction with activities of daily living.

He progressed well and has no limitation of activities of daily living during the latest follow up at age 17 years (Figure 4) and excels in competitive archery.

## Discussion

Congenital constriction band syndrome is a collection of fetal malformations where fibrous bands appear around various fetal parts leading to deformation, malformation, and disruption. These bands can cause anomalies involving limb defects, craniofacial defects, and visceral defects [5]. The pathophysiology of this condition is still not well known, however, there are two theories explaining the possibility of this condition. The intrinsic theory by Streeter in 1930 proposes that these anomalies are a result of perturbation of the developing germinal disc [6]. Torpin in 1965 introduced the extrinsic theory in which these anomalies are caused by fibrous bands in the amniotic cavity [7].

Hall et all in 1982 described three clinical stages of this syndrome; mild, moderate, and severe, depending on the depth of the constriction affecting the underlying lymphatics [8]. The ‘moderate’ stage similar to our case are the cases that require reconstructive surgery. Severe cases would have resulted in intrauterine amputation and mild cases would not require any surgery [8]. The circulation of the limb distal to the constriction usually is not affected by the constriction band due to the endosteal blood supply and myocutaneous arteries [8].

Excision of the constriction bands and reconstruction of the wound remains the mainstay of treatment for moderate and severe stages of congenital constriction band [8]. Multiple stage surgery was initially recommended for treatment of circumferential constriction bands whereby the first half of the band is excised, followed by the other half of the band later [9]. This is done with the aim to prevent disruption to circulation of the limb distal to the band [9]. However, due to the fact that the circulation of the limb distal to the constriction band is supplied by the myocutaneous arteries and endosteal blood supply, single-stage surgery is possible [10]. 

Single-stage surgery with skin reconstruction through Z-plasty offers satisfactory outcomes in terms of wound healing, surgical complications, scar quality, and limb function [4]. A single-stage surgery also eliminates the need for additional operations or anesthesia [11]. Reconstruction of the wound post excision would be either by means of ‘z’ or w-plasty, direct closure, or by rectangular-plasty technique [9,12]. In many previous cases, the decision to reconstruct the wound by z-plasty or w-plasty was to reduce tension during apposition of the wound and also to prevent scar contraction. However, that method was less cosmetically favorable as compared to direct closure [9]. In our patient, the distal ring was scheduled for excision after a suitable interval. This was to allow the proximal wound to heal and the leg edema to subside. We did not proceed with simultaneous excision of both rings as we were uncertain about the safety of this approach.

A previous study had followed up their patients for 10 years to assess their functional outcome [13]. We highlight the outcome after 17 years in our patient, from the neonatal period until skeletal maturity. There was no recurrence of the constriction bands. The lymphoedema resolved as constriction band excision and Z-plasties allowed for the establishment of new lymphatic channels [8]. Our patient had no functional disability.

## Conclusions

Congenital constriction bands causing lymphedema must be treated surgically. We have shown that a single stage excision followed by skin closure with z-plasties is a safe approach with an excellent long-term outcome.
